# Risk prediction models for sarcopenia in elderly people: a systematic review and meta-analysis

**DOI:** 10.3389/fmed.2025.1589583

**Published:** 2025-06-02

**Authors:** Jie Yin, Yiyong Xu, Mian Cai, Xiwei Fang

**Affiliations:** School of Nursing, Jiangxi University of Chinese Medicine, Nanchang, Jiangxi, China

**Keywords:** elderly, sarcopenia, model, prediction, systematic review

## Abstract

**Objectives:**

This study aims to systematically review and evaluate risk prediction models for sarcopenia in older adults. The goal is to offer a reference for clinicians in selecting or developing suitable sarcopenia risk prediction models for the elderly.

**Methods:**

A systematic search was performed across CNKI, Wanfang Database, VIP Database, SinoMed, Embase, PubMed, Web of Science, and Cochrane Library for studies on risk prediction models of sarcopenia in older adults. The time frame for the search was from the creation of these databases to 13 August 2024. The literature was independently vetted by two researchers, who also gathered data and assessed the included studies’ applicability and bias risk.

**Results:**

A total of 29 studies with 70 sarcopenia prediction models were included, with a total sample size of 140,386 and 13,472 sarcopenia events. Frequently reported independent predictors in multivariate models included BMI, age, and gender. Meta-analysis showed a combined AUC of 0.9125 [95% CI (0.9254–0.8996)], indicating good overall model predictive performance. Issues in modeling included inappropriate predictive factor screening methods, insufficient sample sizes, and lack of external validation, resulting in high study bias risk and limited model generalizability.

**Conclusion:**

Current elderly sarcopenia risk prediction models have considerable room for improvement in overall quality and applicability. Future modeling should follow PROBAST guidelines to reduce bias risk, incorporate predictive factors with theoretical foundation and clinical significance, and strengthen external validation.

**Systematic review registration:**

https://www.crd.york.ac.uk/PROSPERO/Diew/CRD42025636116, identifier CRD42025636116.

## 1 Introduction

Sarcopenia is a progressive and systemic disease affecting skeletal muscle, characterized by a gradual loss of muscle mass and function. The main symptoms include muscle weakness and fatigue, with more severe cases leading to difficulties in chewing, muscle atrophy, impaired movement, and muscle numbness (1). Currently, the primary diagnostic criteria for sarcopenia are established by four groups: the Asian Working Group for Sarcopenia (AWGS), the European Working Group on Sarcopenia in Older People (EWGSOP), the International Working Group on Sarcopenia (IWGS), and the Foundation for the National Institute of Health (FNIH) (2–5). Due to the lack of a unified diagnostic standard, reported prevalence rates vary significantly, ranging from 9.9% to 46% (6, 7). Although sarcopenia can occur at any age, it predominantly affects the elderly and substantially contributes to the decline in physiological function. It is associated with an increased risk of mortality, falls, disability, hospitalization, and a reduced ability to live independently (8–11), ultimately impacting daily living and quality of life. This imposes a considerable burden on individuals, society, and the economy. Existing research has found associations with cardiovascular diseases, respiratory system disorders, and cognitive impairments (12–15), making early identification of high-risk populations crucial. The AWGS recommends adopting further strategies to identify sarcopenia-risk patients early, even without advanced diagnostic equipment, and provide timely interventions (3). Predictive models can effectively assist in this regard, leveraging disease predictors to accurately estimate an individual’s likelihood of developing the condition (16). While multiple sarcopenia risk prediction models have been created both nationally and internationally, there is currently no systematic review examining these models. This study aims to systematically review and evaluate risk prediction models for sarcopenia in older people, providing references for model optimization, clinical application, and scientific research.

## 2 Methods

### 2.1 Screening criteria

Inclusion and exclusion criteria were defined according to the Participants, Exposition, Comparators, Outcomes, and Study design (PECOS) framework as follows: (Table 1).

**TABLE 1:** Participants, Exposition, Comparators, Outcomes, and Study design (PECOS) criteria.

Parameters	Inclusion	Exclusion
Participants	Age ≥ 60 years; including community residents, hospital patients, residents of long-term care facilities	Age < 60 years
Exposition	Construction and (or) validation of prediction models for sarcopenia risk in elderly people	No clear definition of sarcopenia diagnosis; only analyzed risk factors for elderly sarcopenia without constructing prediction models; prediction models based on systematic evaluation
Comparators	Not applicable (no specific comparison group)	–
Outcomes	Performance indicators of the constructed sarcopenia risk prediction models (such as AUC, accuracy, sensitivity, specificity, etc.) and the validation and evaluation results of the models	–
Study design	Case-control/cross-sectional/cohort studies	Non-Chinese or English articles; articles with incomplete or inaccessible full text or data information; reviews, conference papers, case reports, Meta-analyses

### 2.2 Search strategy

Computer-based literature retrieval from the China National Knowledge Infrastructure (CNKI), Wanfang Database, VIP Database, SinoMed, Embase, PubMed, Web of Science, and Cochrane Library was conducted. The search period extended from the establishment of these databases to August 13, 2024. Initially, relevant original studies were retrieved from Chinese and English databases, with analysis of titles, keywords, abstracts, and subject terms to further determine search keywords. The search strategy focused on two key concepts: sarcopenia and risk prediction. Search terms included Aged, Elderly, Old, Elder, older adults, sarcopenia, predict*, screening model*, risk, Nomograms, Machine Learning, Risk Assessment, Deep Learning, etc., A combination of free words and subject terms was used, with adjustments made according to the characteristics of each database. (See search strategy document for Supplementary materials).

### 2.3 Literature screening and data extraction

Duplicate literature was removed using EndNote X9. Two researchers independently screened literature and extracted data according to inclusion and exclusion criteria, cross-checking with each other. Disagreements were resolved through discussion or consultation with a third researcher. Literature screening began with reading titles and abstracts, excluding obviously irrelevant literature, followed by full-text reading to determine inclusion. First author, publication year, nation, study design, research participants, sample size, model method, missing data handling method, area under the receiver operating characteristic curve, validation method, and predictive factors were among the data that were extracted.

### 2.4 Quality assessment

Bias risk and applicability were assessed using the Prediction Model Risk of Bias Assessment Tool (PROBAST) (17). Assessments of literature bias risk and applicability were carried out independently by two researchers, with disagreements resolved through discussion with a third researcher. PROBAST includes 20 questions across four domains: participants, predictors, outcomes, and analysis. Assessment results for each domain were judged as low, high, or unclear risk. Each question was answered with yes, probably yes, no, probably no, or unclear. The overall bias risk was considered low if all four domains showed low risk, high if ≥ 1 domain showed high risk, and unclear if ≥ 1 domain was unclear while others showed low risk. Applicability assessment covered three aspects: participants, predictors, and outcomes, following the same evaluation method as bias risk assessment. The applicability of prediction models in each domain and overall was rated as good, poor, or unclear based on corresponding descriptions.

### 2.5 Data analysis

The research in the systematic review and meta-analysis (including specific reasons for exclusion) was summarized using the PRISMA flow chart. The included literature’s essential features, bias risk, and applicability evaluation findings were compiled using descriptive analysis. We extracted the true positive (TP), false positive (FP), false negative (FN), and true negative (TN) values from each study (excluding studies that could not compute 2 × 2 contingency table values and utilizing data from the best model in studies using several models). Meta-analysis was performed using Meta-Disk (v1.4) software to assess the overall performance of the geriatric sarcopenia risk prediction model by plotting SROC curves, sensitivity and specificity forest plots at 95% CI. Heterogeneity analysis was performed using chi-square test and *I*^2^ test, *I*^2^ > 50% indicated significant heterogeneity. Statistical significance was considered *p* < 0.05.

## 3 Results

### 3.1 Literature screening

**FIGURE 1:**
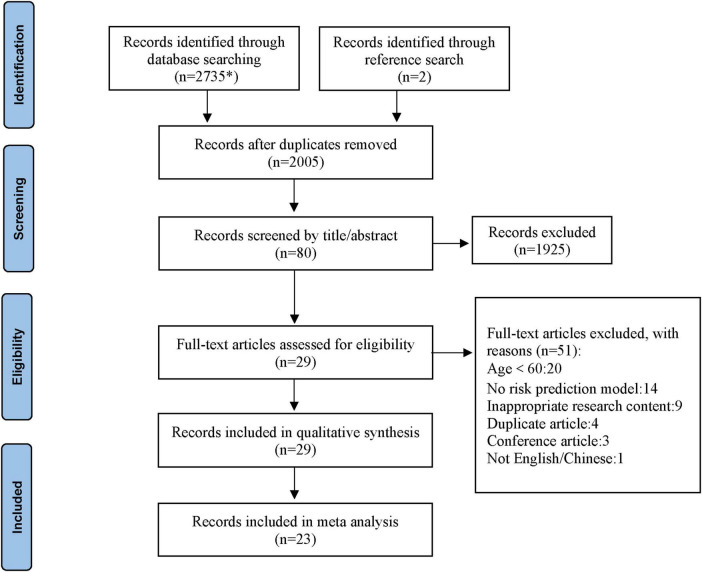
Literature screening process and results. *The databases searched and the number of literature detected were as follows: CNKI (*n* = 351), Wanfang Database (*n* = 359), VIP Database (*n* = 85), SinoMed (*n* = 293), PubMed (*n* = 273), Web of Science (*n* = 618), Embase (*n* = 698), and Cochrane Library (*n* = 58).

Eight Chinese and English databases were searched, yielding 2,737 relevant articles. Using Endnotes X9, 732 duplicate articles were removed, and 1,925 irrelevant articles were eliminated through abstract and title review. Finally, 29 studies (18–46) were included in the systematic review for qualitative analysis, while six studies (25, 26, 34, 36, 38, 44) were excluded from the meta-analysis due to the inability to calculate 2 × 2 contingency table values. Therefore, 23 studies (18–24, 27–33, 35, 37, 39–43, 45, 46) were included in the meta-analysis (Figure 1).

### 3.2 Basic information about the studies

All 29 studies included in the review were published between 2020 and 2024. Mostly consisting of cross-sectional and retrospective studies. China had the most studies (*n* = 19, 18 from mainland China, 1 from Taiwan) (20, 22, 24, 26, 27, 33–46), followed by South Korea (*n* = 6) (18, 23, 25, 29–31), and other countries including the United States (21), Turkey (28), Spain (19), and Iran (32). Research settings were primarily hospitals and communities, along with health database information. Among these, 16 studies (19–21, 23, 24, 28, 29, 35, 37–41, 43–45) were conducted in hospitals, specifically including inpatients (*n* = 12), physical examination patients (*n* = 2), and outpatients (*n* = 2), involving elderly patients with chronic conditions such as stroke, COPD, and diabetes, with three studies (19, 21, 23) focusing on orthopedic patients. 8 studies (22, 27, 32–34, 36, 42, 46) developed sarcopenia prediction models for community-dwelling elderly, while the remaining five studies (18, 25, 26, 30, 31) used elderly data from KNHANES and CHARLS databases (Table 2).

**TABLE 2:** Basic characteristics of the included studies.

References	County	Study design	Regions, setting	Diagnosis of sarcopenia	Sample size	DoS
Bae et al. (18)	Korea	Retrospective	2010–2023 Korean National Physical Fitness Award data	According to the threshold determined by the 20th percentile of ASM/ht^2^; Males: ASM/ht^2^ < 6.54 kg/m^2^, Females: ASM/ht^2^ < 5.14 kg/m^2^	108,304	5,385
Borges et al. (19)	Spain	Cross-sectional	Hip fracture patients; Neurotrau-matology and Rehabilitation Hospital, Granada, Spain	EWGSOP-2	90	27
Cui et al. (20)	China	Retrospective	Type 2 diabetics; The First Hospital of Jilin University	AWGS	132	38
Deer et al. (21)	America	Cross-sectional	Acute Hospitalized Patients; The University of Texas Medical Branch	FNIH/EWGSOP-2	125	30
Huang et al. (22)	China	Cross-sectional	Community-dwelling elderly; Wuhan, Hubei, China	AWGS	966	759
Hwang et al. (23)	Korea	Prospective cohort	Total knee arthroplasty patients; Seoul National University Hospital	AWGS	403	34
Jiang et al. (24)	China	Cross-sectional	Hospitalized patients; Affiliated Kunshan Hospital of Jiangsu University	AWGS	298	151
Kim (25)	Korea	Cross-sectional	KNHANES	AWGS	5573	795
Li et al. (26)	China	Cross-sectional	CHARLS	AWGS; where the cutoff for low muscle mass was determined based on sex-specific criteria. Males: ASM/ht^2^ < 6.88 kg/m^2^ Females: ASM/ht^2^ < 5.69 kg/m^2^	3,454	997
Mo et al. (27)	China	Cross-sectional	Community-dwelling elderly; Hunan, China,	AWGS	1,050	263
Ozgur et al. (28)	Türkiye	Cross-sectional	Geriatrics Clinic Patients; The Ege University Faculty of Medicine	EWGSOP-2	190	28
Ryu et al. (29)	Korea	Prospective cohort	The patients who visited the osteoporosis clinic at Severance Hospital; Seoul, South Korea/The Korean Urban Rural Elderly	AWGS	1,075	159
Seok et al. (30)	Korea	Cross-sectional	KNHANES	AWGS	3,911	1,223
Seok and Kim (31)	Korea	Cross-sectional	KNHANES	AWGS	4,058	1,279
Shafiee et al. (32)	Iran	Prospective cohort	Community-dwelling elderly; Bushehr, South Iran	EWGSOP-2	2,211	505
Tseng et al. (33)	China-Taiwan	Cross-sectional	Community-dwelling elderly; TIHW study	AWGS	1,025	179
Yanget al. (34)	China	Cross-sectional	Community-dwelling elderly; Shaoxing, Zhejiang Province	AWGS	633	125
Yin et al. (35)	China	Cross-sectional	Physical examination patients; Zhongnan Hospital of Wuhan University	AWGS	180	69
Chen et al. (36)	China	Cross-sectional	Community-dwelling elderly; 5 neighborhoods in the old city of Xiangtan	AWGS	556	87
Chen et al. (37)	China	Retrospective	Stroke patients; Nantong Second People’s Hospital	AWGS	80	20
Chen et al. (38)	China	Retrospective	COPD patients; People’s Hospital of Xinjiang Uygur Autonomous Region	AWGS	208	52
Han et al. (39)	China	Cross-sectional	Hospitalized patients; A tertiary grade-A hospital in Urumqi, Xinjiang	SARC-F	695	113
Kong et al. (40)	China	Cross-sectional	Stroke patients, A tertiary grade-A hospital in Liaoning Province	SARC-F	489	184
Li (41)	China	Cross-sectional	Post-operative hip fracture patients; A tertiary grade-A hospital in Zhengzhou City, Henan Province	AWGS	400	151
Liu et al. (42)	China	Cross-sectional	Elderly patients with chronic diseases in the community; A community in Linghe District, Jinzhou City	SARC-F	460	145
Yue et al. (43)	China	Longitudinal	Hospital physical examination patients; A hospital in Shanghai	AWGS	2,544	422
Zhang et al. (44)	China	Cross-sectional	Hospitalized patients; Affiliated Hospital of Qinghai University	AWGS	268	83
Zhang et al. (45)	China	Cross-sectional	Hospitalized patients; The First Affiliated Hospital of Xinjiang Medical University	AWGS	372	70
Zhou (46)	China	Retrospective	Community physical examination patients; 2 community health centers in North District and West Street within Dali City, Dali Prefecture	AWGS	626	99

DoS, development of sarcopenia; KNHANES, Korea National Health and Nutrition Examination Survey; CHARLS, China Health and Retirement Longitudinal Study; TIHW, 2017 Taiwan Health and Welfare study; COPD, chronic obstructive pulmonary disease.

### 3.3 Basic information of the models

#### 3.3.1 Model development

Among the 29 included studies, 11 studies (20, 24, 25, 28–31, 39, 40, 43, 45) developed multiple models to predict the same outcome, totaling 70 sarcopenia prediction models. Most studies (*n* = 28) developed models applicable to both genders. Shafiee et al. (32) only developed prediction models for elderly males and females separately, without providing a model that is applicable to both genders. Reported model performance metrics included model accuracy, sensitivity, specificity, precision, recall, F1 score, and AUC (Table 3, lists only the best model data). Among the modeling methods used, logistic regression was the most frequently employed, with 24 instances, followed by random forest (RF) with 10 instances, and machine learning models such as XGBoost (XGB), LightGBM (LGB), and support vector machines (SVM), which were used 4 times (Figure 4A).

**TABLE 3:** Model performance.

References	Model	Model performance
Bae et al. (18)	DNN	Accuracy = 0.8755, Recall = 0.9075, precision = 0.8523, AUC = 0.9445, F1 = 0.879
Borges et al. (19)	LR	Sensitivity = 0.852, specificity = 0.746, PPV = 0.58, NPV = 0.92, AUC = 0.824
Cui et al. (20)	SVM*	Sensitivity = 0.595, specificity = 0.947, AUC = 0.87
Deer et al. (21)	SMR	Accuracy = 0.88, sensitivity = 0.8, specificity = 0.91, PPV = 0.0.73, NPV = 0.93
Huang et al. (22)	LR	Accuracy = 0.8, sensitivity = 0.94, specificity = 0.781, PPV = 0.369, NPV = 0.99, AUC = 0.921
Hwang et al. (23)	XGB	Accuracy = 0.945, sensitivity = 0.97, specificity = 0.926, AUC = 0.988
Jiang et al. (24)	SVM*	Accuracy = 0.899, sensitivity = 0.657, specificity = 0.840, PPV = 0.872, NPV = 0.912, AUC = 0.775
Kim (25)	LGB*	Accuracy = 0.848, sensitivity = 0.843, specificity = 0.853, PPV = 0.851, NPV = 0.844, AUC = 0.93, F1 score = 0.847
Li et al. (26)	LR	AUC = 0.77
Mo et al. (27)	LR	Sensitivity = 0.681, specificity = 0.825, AUC = 0.827
Ozgur et al. (28)	LGB*	Accuracy = 0.939, Recall = 0.937, precision = 0.943, AUC = 0.984,F1 score = 0.934
Ryu et al. (29)	RF*	Sensitivity = 0.844, specificity = 0.739, precision = 0.397, NPV = 0.959, AUC = 0.813, F1 score = 0.54
Seok et al. (30)	CAT*	Accuracy = 0.790, Recall = 0.792, PPV = 0.792, AUC = 0.868
Seok and Kim (31)	RF*	Accuracy = 0.763, precision = 0.892, Recall = 0.890, AUC = 0.912
Shafiee et al. (32)	LR	Sensitivity = 0.861, specificity = 0.705, PPV = 0.467, NPV = 0.944, AUC = 0.86
Tseng et al. (33)	LR	Sensitivity = 0.718, specificity = 0.711, AUC = 0.757
Yang et al. (34)	LR	AUC = 0.974
Yin et al. (35)	LR	Sensitivity = 0.85, specificity = 0.97, AUC = 0.90, C-index = 0.90
Chen et al. (36)	LR	AUC = 0.895
Chen et al. (37)	LR	Sensitivity = 0.8471, specificity = 0.7988, AUC = 0.826
Chen et al. (38)	LR	Sensitivity = 0.808, specificity = 0.632, AUC = 0.756
Han et al. (39)	LR*	Accuracy = 0.9065, sensitivity = 0.823, specificity = 0.77, AUC = 0.864
Kong et al. (40)	LR*	Accuracy = 0.899, sensitivity = 0.88, specificity = 0.911, PPV = 0.872, NPV = 0.912, AUC = 0.959
Li (41)	LR	Accuracy = 0.8965, sensitivity = 0.91, specificity = 0.8876, PPV = 0.8417, NPV = 0.9375, AUC = 0.928
Liu et al. (42)	LR	Sensitivity = 0.897, specificity = 0.883, AUC = 0.955
Yue et al. (43)	LGB*	Accuracy = 0.896, sensitivity = 0.571, specificity = 0.960, PPV = 0.739, NPV = 0.919, AUC = 0.913
Zhang et al. (44)	LR	C-index = 0.775
Zhang et al. (45)	NN*	Accuracy = 0.858, sensitivity = 0.797, specificity = 0.853, AUC = 0.890
Zhou (46)	LR	Sensitivity = 0.873, specificity = 0.992, AUC = 0.983

*Research that develops multiple models uses data from the best model; LR, logistic regression; NN, neural networks; LGB, Light Gradient Boosting Machine; DNN, deep neural networks; SVM, support vector machine; RF, random forest; CAT, CatBoost; XGB, eXtreme Gradient Boosting; SMR, stepwise multiple regression.

#### 3.3.2 Sample size and predictive factors

**FIGURE 2:**
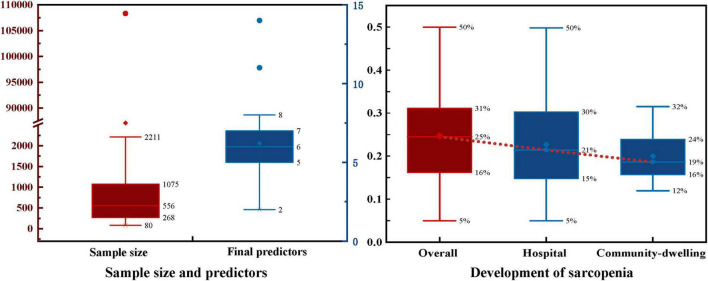
Sample size and development of sarcopenia.

Model development sample sizes ranged from 80 to 108,304 cases, with sarcopenia patient numbers ranging from 27 to 5,385 and sarcopenia prevalence rates from 5% to 50%, averaging 25% (Figure 2). Hospital elderly patients showed higher sarcopenia prevalence than community-dwelling elderly, at 21% and 19%, respectively. Elderly hospital patients often have more underlying diseases, malnutrition, lack of exercise, and other conditions that increase sarcopenia risk.

**FIGURE 3:**
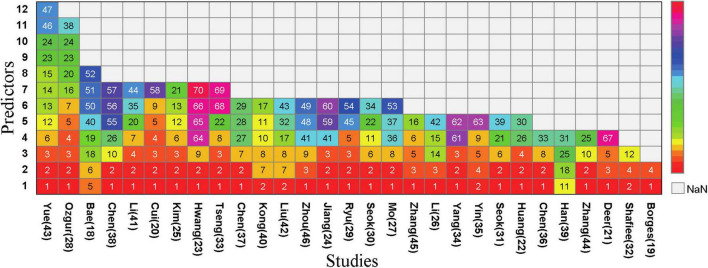
Predictors included in the prediction mode. (1), BMI; (2), Age; (3), Sex; (4), CalfCircumference; (5), Handgrip Strength; (6), Waist Circumference; (7), Nutritional condition; (8), Physical Exercise; (9), Albumin; (10), Smoking; (11), Ability/Quality of Daily Living; (12), Weight; (13), Height; (14), Systolic Blood Pressure; (15), Diastolic Blood Pressure; (16), Gait Speed; (17), Risk of Falling; (18), Sit-and-stand up (counts); (19), 3 m timed up-and-go test; (20), Upper Arm Circumference; (21), Physical Activity; (22), Economic Situation; (23), Presence of Hypertension; (24), Diabetes Mellitus; (25), Osteoporosis; (26), Chronic Obstructive Pulmonary Disease; (27), Stroke; (28), Bone Density; (29), Hyperlipidemia; (30), Congestive Heart Failure; (31), Chronic Bronchitis; (32), Number of Diseases; (33), Thigh Circumference; (34), Social Infrastructure Facilities; (35), Level of Education; (36), Marital Status; (37), Dietary Diversity; (38), SARC-F; (39), International Physical Activity Questionnaire; (40), Body Fat; (41), Total Cholesterol; (42), Pain; (43), Fatigue; (44), Skeletal Muscle Index; (45), Appendicular Lean Mass; (46), Hip Circumference; (47), Heart Rate; (48), Exercise Frequency; (49), exercise Duration; (50), Sit-and-reach (cm); (51), 2 min step (counts); (52), Figure-of-8 walk (sec); (53),Sedentary Time; (54),Chair Rise Test; (55), FEV1/FVC%; (56), Number of Annual Acute Exacerbations of COPD; (57), COPD Assessment Test TM; (58), 25-Hydroxyvitamin D3; (59),Triglyceride; (60), Homocysteine; (61),uric acid; (62), alanine aminotransferase; (63),blood urea nitrogen; (64), Hemoglobin; (65), bilirubin; (66), predicted muscle volume; (67), BIA measured FM%; (68), Blood glucose; (69), creatinine; (70), total protein.

**FIGURE 4:**
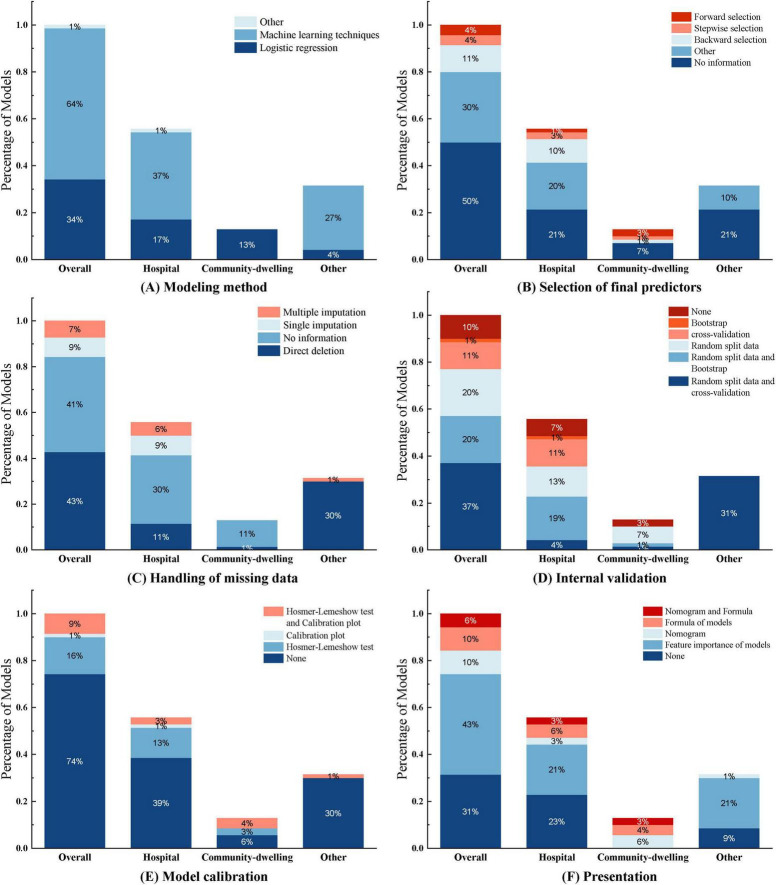
This figure illustrates the construction of the 70 models included in the study across different aspects. Each subfigure **(A–F)** consists of four categories: Overall, Hospital, Community-dwelling, and Other, representing the distribution of models under different application environments. **Overall:** Represents the overall distribution of all 70 research models, covering all application environments and scenarios. **Hospital:** Represents the distribution of models applied in hospital environments. **Community-dwelling:** Represents the distribution of models applied in community dwelling environments. **Other:** Represents the distribution of models applied in other environments (such as database construction).

Half of the models (*n* = 35, 50%) did not report any method for the selection of final predictive variables, eight models reported using backward selection, and three models each (4.2%) used forward selection and stepwise selection methods for choosing the final predictive factors. Additionally, some models (*n* = 21, 30%) used other methods such as lasso selection, principal component analysis, empirical selection, etc., (Figure 4B). The median number of final predictive variables (min, max) was six (2, 14). Evaluating sample size reasonability based on the Events Per Variable (EPV) ratio, only five studies (18, 25, 30–32) had EPV values greater than 20. The top three repeatedly reported independent predictors in multivariate models were BMI (*n* = 24), age (*n* = 22), and gender (*n* = 13) (Figure 3).

#### 3.3.3 Handling of missing data

Among the 70 included models, 29 models (41.4%) did not report any method for handling missing data, nearly half of the models (*n* = 30, 42.9%) reported directly excluding subjects with incomplete records, six models (8.6%) used single imputation methods, and only five models (7.1%) used multiple imputation methods to handle missing data (Figure 4C).

#### 3.3.4 Internal and external validation

Common internal validation methods include random split data, cross-validation, and bootstrap sampling. In this study, 26 models (37.1%) used random split data and cross-validation, 14 models (20%) used random split data and bootstrap for internal validation, 14 models (20%) only used random split data, 8 models (11.4%) only used cross-validation, and one model (1%) only used bootstrap sampling for internal validation (Figure 4D). External validation typically employs different time periods, different study locations, or completely independent datasets to test the model’s predictive performance in external environments. In this study, only six models (8.6%) underwent external validation, among which the LR model constructed by Chen Xi et al. (38) used a dataset from the same location but different time period for external validation. Notably, seven models (10%) did not undergo any internal or external validation; they were merely constructed without validation.

#### 3.3.5 Model performance evaluation and presentation

Model calibration was performed using the Hosmer-Lemeshow test and calibration plots. Most models (*n* = 52, 74.3%) did not report any calibration assessment method. 11 models (15.7%) used the Hosmer-Lemeshow test, with *p*-values all greater than 0.05, indicating good agreement between the models and observed values. Only six models (8.6%) used both the Hosmer-Lemeshow test and calibration plots for model calibration (Figure 4E).

The model performance assessment reported in the study involved 68 models using ROC curves with AUCs ranging from 0.706 to 0.995. The LR model constructed by Zhang et al. (44) only used the C-index, which was 0.775. Deer et al. (21) did not report ROC curves or C-index in their stepwise multivariate regression model for elderly acute hospital patients.

Regarding model presentation, among the 25 regression models in this study, seven models (10%) used nomograms for visual presentation, seven models (10%) constructed regression-based equations, four models (5.7%) used both nomograms and equations, and seven models (10%) did not provide any representation format. Of the 45 sarcopenia models built using machine learning, most models (*n* = 30, 42.9%) ranked variable feature importance (Figure 4F). (See Supplementary Table 1).

### 3.4 Risk of bias assessment

**FIGURE 5:**
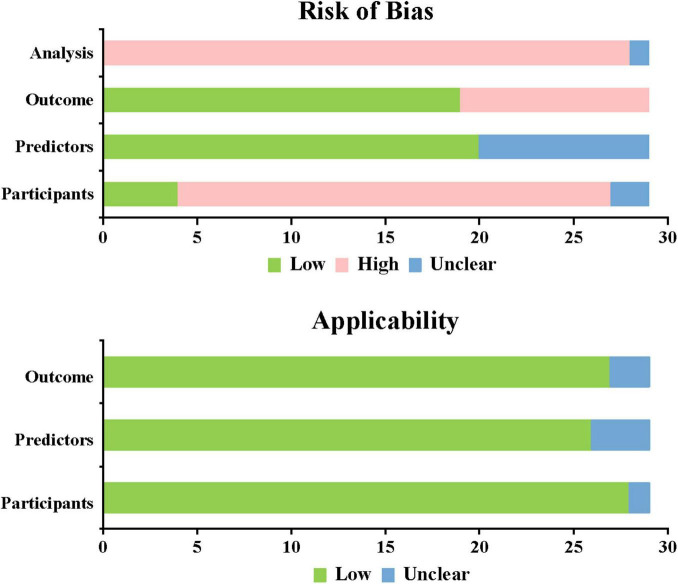
Risk of bias and applicability assessment.

This study used PROBAST, which provides a standardized assessment framework for evaluating prediction model studies. The overall assessment showed high bias risk but good applicability for all included studies. All 29 included studies showed high overall bias risk. 26 studies (18–28, 30, 31, 33–42, 44–46) based on cross-sectional and retrospective research were at high bias risk. A total of nine studies (20, 21, 28, 29, 35, 40–42, 45) were unclear in predictive factors, and 10 studies (20, 21, 28, 29, 32, 35, 40–42, 45) were at high risk in predictive outcomes, while other studies showed lower risk. In the analysis domain, 28 studies (18–26, 28–46) were at high risk and one study (27) was unclear. Among these, 17 studies (18–20, 22–25, 27, 28, 30–33, 36, 40, 41) had EPV < 20, indicating unreasonable sample sizes; 25 studies (18, 19, 21–25, 27–34, 36–42, 44–46) did not properly handle missing data; 16 studies (18–20, 23, 25, 28–32, 38–41, 43, 45) were at high risk due to univariate selection of predictive variables (Figure 5). (See Supplementary Table 1).

### 3.5 Meta analysis

**FIGURE 6:**
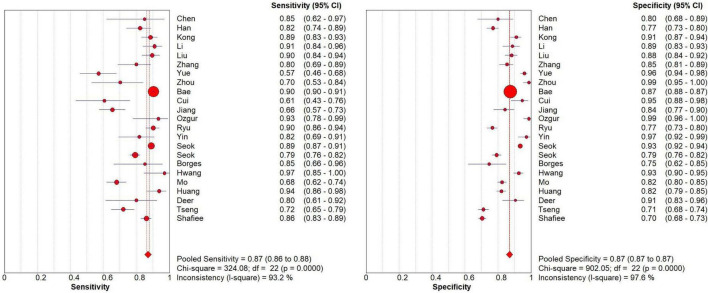
Meta-analysis of models.

Meta-analysis of prediction models based on 2 × 2 contingency tables showed no threshold effect heterogeneity (Spearman analysis *p*-value = 0.922, *p* ≥ 0.05). Using the random effects model, the combined sensitivity, specificity, and diagnostic odds ratio were 0.87 [95% CI (0.86–0.88)], 0.87 [95% CI (0.87–0.87)], and 37.38 [95% CI (24.02–58.18)], respectively. The SROC curve was plotted to assess model accuracy, with a combined AUC of 0.9125 [95% CI (0.9254–0.8996)], indicating high model accuracy. Heterogeneity analysis of the I2 statistic showed a high degree of heterogeneity between studies (*p* < 0.0001). A funnel plot analysis was used to assess publication bias, and it showed a *p*-value of 0.24, indicating the presence of publication bias (Figure 6, Supplementary Figures 1–3).

## 4 Discussion

The analysis included 29 studies on sarcopenia prediction models, which reported an average prevalence rate of 25%, with rates ranging from 5% to 50%. The findings indicate a significant prevalence of sarcopenia among older adults. Relevant risk prediction models can aid healthcare professionals in the early identification of high-risk populations and in implementing preventive interventions to reduce the incidence of sarcopenia. Although several sarcopenia prediction models for older individuals have been reported, a systematic review of these models has not yet been conducted. This study aims to evaluate the effectiveness of existing sarcopenia prediction models through a systematic review and meta-analysis, providing a reference for the development and enhancement of sarcopenia risk prediction models.

This study examined 70 sarcopenia models, with AUC values ranging from 0.706 to 0.995. Meta-analysis of the models yielded a combined AUC of 0.9125 [95% CI (0.9254–0.8996)], indicating good discriminative ability of the included models. However, heterogeneity analysis revealed high heterogeneity among studies, possibly due to differences in study design, subjects, models, and sarcopenia diagnostic criteria. PROBAST quality assessment showed high overall applicability of included studies but a high risk of bias, with issues primarily in sample source, clinical outcomes, and statistical analysis. (1) Regarding sample sources, most of the literature included was based on cross-sectional and retrospective studies, which lacked sample representativeness. (2) Regarding clinical outcomes, some studies incorporated sarcopenia diagnostic factors like grip strength and walking speed as final predictive variables, potentially overestimating model performance and creating merger bias. (3) Regarding statistical analysis: ① Sample size, judged by EPV criteria (47). PROBAST (17) suggests that EPV ≥ 20 can reduce bias risk; 24 studies in this research had high sample size bias risk. ② Missing data handling: detailed explanation and proper handling of missing data help prevent model overfitting (48). Among 70 models, 29 did not report handling methods, 30 directly eliminated incomplete data, and only five used multiple imputations. ③ Predictive factors: most studies determined final predictive variables through univariate analysis and logistic regression. Yin et al. (35) enhanced model practicality by removing difficult-to-obtain BMI indicators for bedridden elderly. ④ Model validation: external validation better enhances model generalizability and cost-effectiveness than internal validation (49). In this study, only the five models constructed by Ryu et al. (29) were simultaneously subjected to internal and external validation.

The study identified 71 predictive factors, with BMI (*n* = 24), age (*n* = 22), and gender (*n* = 13) appearing more than 10 times. Research indicates that after age 50, muscle strength declines by 1.5% to 5% annually, while muscle mass decreases by 1%–2% each year (50). Sarcopenia incidence increases significantly with age (51). Among elderly populations, various aging-related factors can lead to sarcopenia development. Mitochondrial dysfunction, protein synthesis and degradation imbalance, cell apoptosis, hormonal disorders, and inflammatory responses can all cause decline in muscle fiber quality, strength, and endurance, inducing sarcopenia development (52, 53). Additionally, aging-related reduced physical activity exacerbates muscle mass loss (54). Currently, there are gender differences in the occurrence, development, and related risk factors of sarcopenia. These differences may stem from multiple factors: On one hand, variations in research subject selection, diagnostic criteria definition, and assessment methods can lead to different incidence rates of sarcopenia; on the other hand, gender-specific physiological characteristics also play an important role. Testosterone and insulin-like growth factor-1 (IGF-1) are crucial for muscle growth and repair (55, 56). During the aging process in men, the rapid decline in testosterone and IGF-1 levels leads to significant decreases in muscle strength and mass. In women, ovarian failure during perimenopause and the decrease in serum estradiol levels can cause abnormal activation of the immune system, thereby accelerating the occurrence and development of sarcopenia (57). Elderly women during this period experienced a faster growth rate in the incidence of sarcopenia. There are also gender differences in gene expression related to sarcopenia. Research by GAO et al. (58) found that when men enter old age, the genes with altered expression during sarcopenia development are more concentrated, whereas the biological mechanisms involved in women are more complex. Additionally, The relationship between sarcopenia and BMI is complex and influenced by multiple factors. Wang et al. (59) found a U-shaped relationship between BMI and sarcopenia occurrence, where both low and high BMI may increase sarcopenia risk. One study (60) analyzing the Irish Longitudinal Study on Aging data found a significant correlation between low BMI and sarcopenia occurrence, while for overweight or obese populations, results varied with different sarcopenia definitions (e.g., lower limb strength and hand grip strength). Low BMI may reflect malnutrition, while obesity and sarcopenia interact through complex pathophysiological mechanisms involving pro-inflammatory cytokines, oxidative stress, insulin resistance, and reduced physical activity. In obese populations, a high BMI may overshadow decreased muscle mass due to increased fat tissue, resulting in sarcopenic obesity (61). Research shows that patients with abdominal obesity (increased visceral fat area VFA) have significantly increased sarcopenia risk even with normal BMI (62). Additionally, some studies indicate higher BMI as a protective factor against sarcopenia (63, 64). Therefore, sarcopenia assessment and management require careful consideration of BMI and other related factors.

The 29 studies included in this research were all published within the past 5 years, indicating a growing interest in clinical risk prediction models. Numerous sarcopenia prediction models have been reported domestically and internationally, primarily targeting community and hospital elderly patients, with most models showing high predictive performance. However, issues exist in modeling predictive factor selection and screening, such as insufficient sample size, improper handling of missing data, and high study bias risk. Therefore, future research could optimize in the following aspects: (1) Use cohort studies, randomized controlled trials, or nested case-control or case-cohort study designs to reduce subject bias risk. (2) Determine candidate predictive variables through literature review, statistical analysis, and expert opinion to ensure comprehensiveness and scientific validity of predictive factors. (3) Include multi-center, large-sample studies, using EPV ≥ 20 or machine learning requirements for sample size calculation. This helps identify model performance in different populations and provides the basis for model adjustment. (4) Avoid univariate variable screening and adjust according to clinical reality to enhance model practicality. (5) Choose different imputation methods for handling missing data based on data type. (6) To enhance the robustness and generalizability of the model, it is important to integrate both internal and external validation methods.

There are several restrictions on this study: ① Only Chinese and English literature were included, creating publication bias; ② Included sarcopenia risk prediction models had high bias risk; ③ Six papers were excluded from meta-analysis due to insufficient data, potentially affecting results.

## 5 Conclusion

In conclusion, this systematic review of elderly sarcopenia risk prediction models found that while most models showed good discriminative ability, they had high overall bias risk and limited generalizability. For future modeling, it is recommended to follow PROBAST guidelines to reduce bias risk, incorporate predictive factors with theoretical foundation and clinical significance, and strengthen external validation to enhance the clinical application of prediction models.

## Data Availability

The original contributions presented in this study are included in this article/Supplementary material, further inquiries can be directed to the corresponding author.
