# Supplementary material for: Risk prediction models for sarcopenia in elderly people: a systematic review and meta-analysis
Source: Front Med (Lausanne). 2025 Jun 2;12:1589583. doi: 10.3389/fmed.2025.1589583 (PMC12171125; doi:10.3389/fmed.2025.1589583)
Supplement: Supplementary file 1 [file Data_Sheet_1.zip › Supplementary Material/Scope statement .docx]

**Scope statement:** This systematic review and meta-analysis focuses on evaluating risk prediction models for sarcopenia in elderly populations, a critical area of geriatric medicine. Sarcopenia, characterized by progressive loss of muscle mass and function, significantly impacts the health and quality of life of older adults. This study synthesizes existing evidence on predictive tools, assessing their accuracy, applicability, and clinical utility. By identifying gaps and strengths in current models, this work aims to provide scientific evidence for the development and optimization of future sarcopenia risk prediction models, promoting the improvement of risk assessment models and offering more precise evaluation tools for clinical practice. This study is highly relevant to《*Frontiers in Medicine*》, as they align with the journal's focus on advancing clinical practice and addressing public health challenges, particularly in aging populations.
