# Supplementary material for: Risk prediction models for sarcopenia in elderly people: a systematic review and meta-analysis
Source: Front Med (Lausanne). 2025 Jun 2;12:1589583. doi: 10.3389/fmed.2025.1589583 (PMC12171125; doi:10.3389/fmed.2025.1589583)
Supplement: Supplementary file 1 [file Data_Sheet_1.zip › Supplementary Material/Search strategy.docx]

**Search strategy**

**A combined subject and free word search was conducted using the following Chinese and English databases: China National Knowledge Infrastructure (CNKI), Wanfang Database, VIP Database, SinoMed, Embase, PubMed, Web of Science, and Cochrane Library****. Taking PubMed as an example:**

| **PubMed** **(from the inception to August 13th, 2024), n=273** | |
| --- | --- |
| **#1** | "Nomograms"[MeSH Terms] OR "Artificial Intelligence"[MeSH Terms] OR "Machine Learning"[MeSH Terms] OR "Risk Assessment"[MeSH Terms] OR "Deep Learning"[MeSH Terms] OR "Decision Trees"[MeSH Terms] OR "neural networks, computer"[MeSH Terms] OR "Support Vector Machine"[MeSH Terms] OR "Bayes Theorem"[MeSH Terms] OR "Random Forest"[MeSH Terms] |
| **#2** | "Sarcopenia"[MeSH Terms] |
| **#3** | "Aged"[MeSH Terms] |
| **#4** | "Aged"[Title/Abstract] OR "Elderly"[Title/Abstract] OR "Old"[Title/Abstract] OR "Elder"[Title/Abstract] OR "Senior"[Title/Abstract] OR "older adults"[Title/Abstract] OR "grey hair"[Title/Abstract] |
| **#5** | "Sarcopenia"[Title/Abstract] OR "Sarcopenias"[Title/Abstract] |
| **#6** | "Nomograms"[Title/Abstract] OR "Nomogram"[Title/Abstract] OR "partin tables"[Title/Abstract] OR "partin nomograms"[Title/Abstract] OR "partin nomogram"[Title/Abstract] OR "random forest*"[Title/Abstract] OR "k nearest neighbour"[Title/Abstract] OR "artificial intelligence"[Title/Abstract] OR "computational intelligence"[Title/Abstract] OR "machine intelligence"[Title/Abstract] OR "computer reasoning"[Title/Abstract] OR "computer vision systems"[Title/Abstract] OR "computer vision system"[Title/Abstract] OR "machine learning"[Title/Abstract] OR "transfer learning"[Title/Abstract] OR "risk assessment"[Title/Abstract] OR "risk assessments"[Title/Abstract] OR "risk analysis"[Title/Abstract] OR "risk analyses"[Title/Abstract] OR "deep learning"[Title/Abstract] OR "hierarchical learning"[Title/Abstract] OR "decision trees"[Title/Abstract] OR "decision tree"[Title/Abstract] OR "neural networks computer"[Title/Abstract] OR "support vector machine"[Title/Abstract] OR "support vector network"[Title/Abstract] OR "bayes theorem"[Title/Abstract] OR "random forest"[Title/Abstract] OR "random forests"[Title/Abstract] OR "random forest classification"[Title/Abstract] |
| **#7** | "prediction model"[Title/Abstract] OR "predict*"[Title/Abstract] |
| **#8** | **#1** OR **#6** |
| **#9** | **#2** OR **#5** |
| **#10** | **#3** OR**#4** |
| **#11** | **#7** AND **#8** AND **#9** AND **#10** |
