# Supplementary material for: Risk prediction models for sarcopenia in elderly people: a systematic review and meta-analysis
Source: Front Med (Lausanne). 2025 Jun 2;12:1589583. doi: 10.3389/fmed.2025.1589583 (PMC12171125; doi:10.3389/fmed.2025.1589583)
Supplement: Supplementary file 1 [file Data_Sheet_1.zip › Supplementary Material/Supplementary Figures.docx]

Supplementary Material

# Supplementary Figures

## Supplementary Figures

**Supplementary Figure 1.**


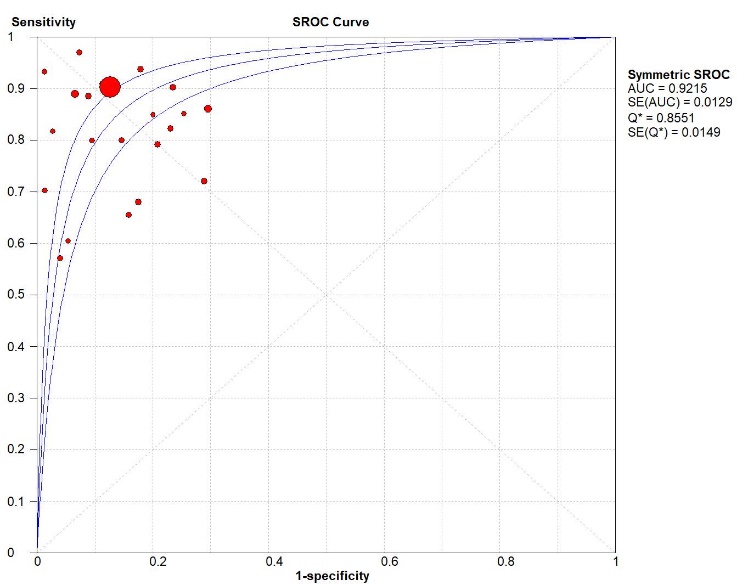


Figure.1 ROC curves for predictive models of sarcopenia

**Supplementary Figure 2.**


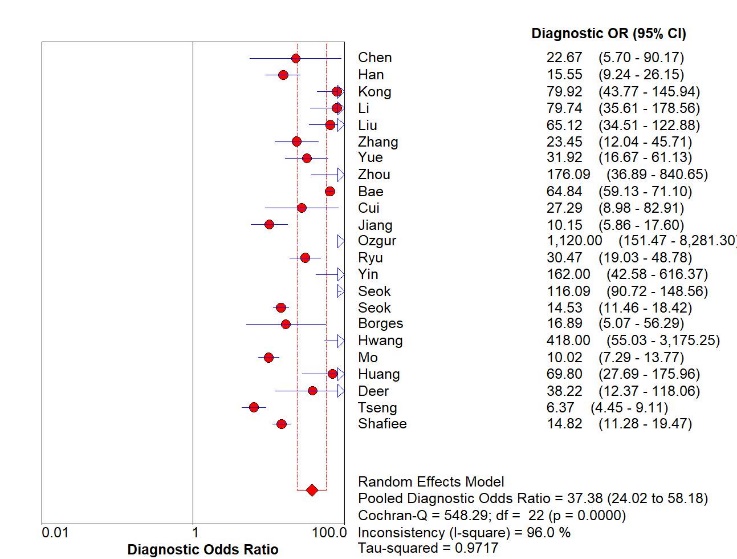


Figure.2 Diagnostic Odds Ratio of Predictive Models for Sarcopenia

**Supplementary Figure 3.**


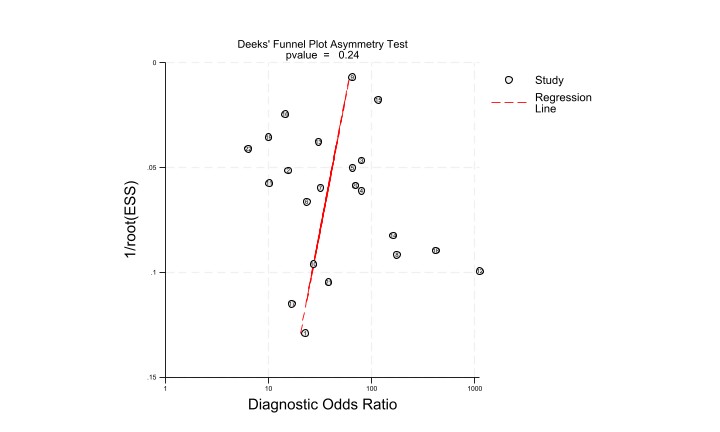


Figure.3 Publication bias of studies
