# Supplementary material for: Risk prediction models for sarcopenia in elderly people: a systematic review and meta-analysis
Source: Front Med (Lausanne). 2025 Jun 2;12:1589583. doi: 10.3389/fmed.2025.1589583 (PMC12171125; doi:10.3389/fmed.2025.1589583)
Supplement: Supplementary file 1 [file Data_Sheet_1.zip › Supplementary Material/Supplementary Table 1.docx]

**Supplementary Table 1**

**Basic information of the included predictive models**

| Numbers | Author, Year | Modelling method | Model discrimination | Handling of  missing data | Selection of  final predictors | Type of validation | Model calibration | Presentation |
| --- | --- | --- | --- | --- | --- | --- | --- | --- |
| 1 | Jiawei Chen,2023 | Logistic Regression | AUC:0.895(0.859-0.931) | No information | No information | Int: None Ext : None | None | Nomogram |
| 2 | Linlin Chen,2023 | Logistic Regression | AUC:0.826 (0.785-0.893) | No information | No information | Int: None Ext : None | Hosmer-Lemeshow test | Formula |
| 3 | Xi Chen,2023 | Logistic Regression | AUC:0.756 (0.673-0.839) | No information | No information | Int: None Ext : Completely independent | Hosmer-Lemeshow test | Formula |
| 4 | Ting Han,2022 | Logistic Regression | AUC:0.864 (0.829-0.899) | No information | No information | Int: None Ext : None | Hosmer-Lemeshow test | None |
| 5 | Ting Han,2022 | Decision Tree | AUC:0.779 (0.756-0.852) | No information | No information | Int: None Ext : None | Hosmer-Lemeshow test | None |
| 6 | Linghui Kong,2024 | Logistic Regression | AUC:0.959(0.943-0.975) | No information | No information | Int: Random split data Ext : None | Hosmer-Lemeshow test | Formula and Nomogram |
| 7 | Linghui Kong,2025 | Decision Tree | AUC:0.892(0.853-0.928) | No information | No information | Int: Random split data Ext : None | Hosmer-Lemeshow test | None |
| 8 | Huijing Li,2023 | Logistic Regression | AUC:0.928(0.900-0.957) | Direct deletion | Forward selection | Int: Random split data  and Bootstrap Ext : None | Hosmer-Lemeshow test and Calibration plot | Formula and Nomogram |
| 9 | Yanping Liu,2022 | Logistic Regression | AUC:0.955(0.937 -0.973) | No information | No information | Int: None Ext : None | Hosmer-Lemeshow test and Calibration plot | Formula and Nomogram |
| 10 | Yuan Zhang,2023 | Logistic Regression | AUC:0.882(0.836 -0.928) | No information | No information | Int: Random split data Ext : None | Hosmer-Lemeshow test | Formula |
| 11 | Yuan Zhang,2023 | Decision Tree | AUC:0. 874(832 -0.916) | No information | No information | Int: Random split data Ext : None | Hosmer-Lemeshow test | None |
| 12 | Yuan Zhang,2023 | Neural Networks | AUC:0.890(0.848-0.931) | No information | No information | Int: Random split data Ext : None | Hosmer-Lemeshow test | Feature importance of models |
| 13 | Yibing Yue,2023 | Logistic Regression | AUC:0.897 | InBody deduplication and k-Nearest-Neighbor | Other | Int: Random split data and Cross-validation Ext : None | None | Feature importance of models |
| 14 | Yibing Yue,2023 | Random Forest | AUC:0.9 | InBody deduplication and k-Nearest-Neighbor | Other | Int: Random split data and Cross-validation Ext : None | None | Feature importance of models |
| 15 | Yibing Yue,2023 | LightGBM | AUC:0.913 | InBody deduplication and k-Nearest-Neighbor | Other | Int: Random split data and Cross-validation Ext : None | None | Feature importance of models |
| 16 | Ying Zhang,2020 | Logistic Regression | C-index:0.775 | No information | Stepwise selection | Int: Bootstrap Ext : None | Calibration plot | Nomogram |
| 17 | Mengjuan Zhou,2023 | Logistic Regression | AUC:0.983(0.971-0.995) | No information | No information | Int: Random split data Ext : None | Hosmer-Lemeshow test and Calibration plot | Formula and Nomogram |
| 18 | Jun-Hyun Bae,2023 | Deep Neural Networks | AUC:0.9445 | Direct deletion | Other | Int: Random split data and Cross-validation Ext : None | None | Feature importance of models |
| 19 | Mengzhao Cui,2020 | Support Vector Machine(3) | AUC:0.85 (0.84-0.86) | k-Nearest-Neighbor | Backward selection | Int: Leave one out and Cross-validation Ext : None | None | None |
| 20 | Mengzhao Cui,2020 | Random Forest(3) | AUC: 0.76(0.75-0.77 ) | k-Nearest-Neighbor | Backward selection | Int: Leave one out and Cross-validation Ext : None | None | None |
| 21 | Mengzhao Cui,2020 | Support Vector Machine(5) | AUC:0.87(0.76-0.98) | k-Nearest-Neighbor | Backward selection | Int: Leave one out and Cross-validation Ext : None | None | None |
| 22 | Mengzhao Cui,2020 | Random Forest(5) | AUC:0.810(0.802-0.890) | k-Nearest-Neighbor | Backward selection | Int: Leave one out and Cross-validation Ext : None | None | None |
| 23 | Mengzhao Cui,2020 | Support Vector Machine(7) | AUC:0.87(0.80-0.94) | k-Nearest-Neighbor | Backward selection | Int: Leave one out and Cross-validation Ext : None | None | None |
| 24 | Mengzhao Cui,2020 | Random Forest(7) | AUC:0.85(0.77-0.93) | k-Nearest-Neighbor | Backward selection | Int: Leave one out and Cross-validation Ext : None | None | None |
| 25 | Yiwen Jiang,2023 | Logistic Regression | AUC:0.764 (0.659–0.869) | No information | Other | Int: Random split data Ext : None | None | None |
| 26 | Yiwen Jiang,2023 | Support Vector Machine | AUC:0.775 (0.670–0.880) | No information | Other | Int: Random split data Ext : None | None | None |
| 27 | Yiwen Jiang,2023 | K-Nearest Neighbors | AUC:0.748 (0.643–0.853) | No information | Other | Int: Random split data Ext : None | None | None |
| 28 | Yiwen Jiang,2023 | XGBoost | AUC:0.706 (0.596–0.816) | No information | Other | Int: Random split data Ext : None | None | None |
| 29 | Qiugui Li,2024 | Logistic Regression | AUC:0.77 (0.75-0.79) | Multiple imputation | Other: LASSO selection | Int: Random split data and Cross-validation Ext: None | Hosmer-Lemeshow test and Calibration plot | Nomogram |
| 30 | Su Ozgur,2023 | LightGBM | AUC:0.984 | Direct deletion | Other | Int: Random split data and Bootstrap Ext : None | None | None |
| 31 | Su Ozgur,2023 | Support Vector Machine | AUC:0.995 | Direct deletion | Other | Int: Random split data and Bootstrap Ext : None | None | None |
| 32 | Su Ozgur,2023 | Random Forest(Both) | AUC:0.949 | Direct deletion | Other | Int: Random split data and Bootstrap Ext : None | None | None |
| 33 | Su Ozgur,2023 | Random Forest(Female) | AUC:0.972 | Direct deletion | Other | Int: Random split data and Bootstrap Ext : None | None | None |
| 34 | Su Ozgur,2023 | XGBoost | AUC:0.941 | Direct deletion | Other | Int: Random split data and Bootstrap Ext : None | None | None |
| 35 | Su Ozgur,2023 | K-Nearest Neighbors | AUC:0.966 | Direct deletion | Other | Int: Random split data and Bootstrap Ext : None | None | None |
| 36 | Jin Ryu,2023 | Random Forest | AUC:0.813 | No information | No information | Int: Random split data and Bootstrap Ext : Completely independent | None | None |
| 37 | Jin Ryu,2023 | Logistic Regression | AUC:0.785 | No information | No information | Int: Random split data and Bootstrap Ext : Completely independent | None | None |
| 38 | Jin Ryu,2023 | XGBoost | AUC:0.787 | No information | No information | Int: Random split data and Bootstrap Ext : Completely independent | None | None |
| 39 | Jin Ryu,2023 | Extra Trees Classifie | AUC:0.795 | No information | No information | Int: Random split data and Bootstrap Ext : Completely independent | None | None |
| 40 | Jin Ryu,2023 | LightGBM | AUC:0.786 | No information | No information | Int: Random split data and Bootstrap Ext : Completely independent | None | None |
| 41 | Yichen Yang,2023 | Logistic Regression | AUC:0.974(0.962–0.987) | No information | Forward selection | Int: Random split data  Ext : None | Hosmer-Lemeshow test | Nomogram |
| 42 | Guangjiao Yin,2023 | Logistic Regression | AUC:0.90(0.85-0.95)  C-index:0.90 | Multiple imputation | Other: LASSO selection | Int: Random split data and Bootstrap Ext : None | Hosmer-Lemeshow test and Calibration plot | Nomogram |
| 43 | Jun-hee Kim,2024 | Logistic Regression | AUC:0.85 | Direct deletion | Other | Int: Random split data and Cross-validation Ext : None | None | Feature importance of models |
| 44 | Jun-hee Kim,2024 | Support Vector Machine | AUC:0.87 | Direct deletion | Other | Int: Random split data and Cross-validation Ext : None | None | None |
| 45 | Jun-hee Kim,2024 | XGBoost | AUC:0.92 | Direct deletion | Other | Int: Random split data and Cross-validation Ext : None | None | Feature importance of models |
| 46 | Jun-hee Kim,2024 | LightGBM | AUC:0.93 | Direct deletion | Other | Int: Random split data and Cross-validation Ext : None | None | Feature importance of models |
| 47 | Jun-hee Kim,2024 | Random Forest | AUC:0.91 | Direct deletion | Other | Int: Random split data and Cross-validation Ext : None | None | Feature importance of models |
| 48 | Jun-hee Kim,2024 | Multi-layer Perceptron | AUC:0.86 | Direct deletion | No information | Int: Random split data and Cross-validation Ext : None | None | None |
| 49 | Minje Seok,2023 | Gradient Boosting Machine | AUC:0.86 | Direct deletion | No information | Int: Random split data and Cross-validation Ext : None | None | Feature importance of models |
| 50 | Minje Seok,2023 | XGBoost | AUC:0.91 | Direct deletion | No information | Int: Random split data and Cross-validation Ext : None | None | Feature importance of models |
| 51 | Minje Seok,2023 | LightGBM | AUC:0.897 | Direct deletion | No information | Int: Random split data and Cross-validation Ext : None | None | Feature importance of models |
| 52 | Minje Seok,2023 | CatBoost | AUC:0.888 | Direct deletion | No information | Int: Random split data and Cross-validation Ext : None | None | Feature importance of models |
| 53 | Minje Seok,2023 | Logistic Regression | AUC:0.839 | Direct deletion | No information | Int: Random split data and Cross-validation Ext : None | None | None |
| 54 | Minje Seok,2023 | K-Nearest Neighbors | AUC:0.791 | Direct deletion | No information | Int: Random split data and Cross-validation Ext : None | None | None |
| 55 | Minje Seok,2023 | Support Vector Classifier | AUC:0.839 | Direct deletion | No information | Int: Random split data and Cross-validation Ext : None | None | None |
| 56 | Minje Seok,2023 | Random Forest | AUC:0.912 | Direct deletion | No information | Int: Random split data and Cross-validation Ext : None | None | Feature importance of models |
| 57 | Minje Seok,2023 | Multi-layer Perceptron | AUC:0.844 | Direct deletion | No information | Int: Random split data and Cross-validation Ext : None | None | None |
| 58 | Minje Seok,2023 | Deep Neural Networks | AUC:0.869 | Direct deletion | No information | Int: Random split data and Cross-validation Ext : None | None | Feature importance of models |
| 59 | Minje Seok,2023 | LightGBM | AUC:0.856 | Direct deletion | No information | Int: Random split data and Cross-validation Ext : None | None | Feature importance of models |
| 60 | Minje Seok | CatBoost | AUC:0.868 | Direct deletion | No information | Int: Random split data and Cross-validation Ext : None | None | Feature importance of models |
| 61 | Minje Seok | Random Forest | AUC:0.85 | Direct deletion | No information | Int: Random split data and Cross-validation Ext : None | None | Feature importance of models |
| 62 | Minje Seok | Deep Neural Networks | AUC:0.817 | Direct deletion | No information | Int: Random split data and Cross-validation Ext : None | None | Feature importance of models |
| 63 | Keith Borges,2022 | Logistic Regression | AUC:0.824(0.746–0.853) | No information | Backward selection | Int: None Ext : None | None | None |
| 64 | Doohyun Hwang,2022 | XGBoost | AUC:0.988(0.986–0.989) | No information | No information | Int: Cross-validation Ext : None | None | Feature importance of models |
| 65 | Yi-Han Mo,2022 | Logistic Regression | AUC:0.827(0.792-0.860) | No information | Backward selection | Int: Random split data and Cross-validation Ext : None | Hosmer-Lemeshow test and Calibration plot | Nomogram |
| 66 | Shuai-Wen Huang,2023 | Logistic Regression | AUC:0.930(0.907-0.952) | Direct deletion | Stepwise selection | Int: Random split data and Bootstrap Ext : None | Hosmer-Lemeshow test | Nomogram |
| 67 | Rachel R. Deer,2020 | Stepwise Multiple Regression | No information | Direct deletion | Stepwise selection | Int: Cross-validation Ext : None | None | Formula |
| 68 | Tzyy-Guey Tseng,2020 | Logistic Regression | AUC:0.757 | No information | Forward selection | Int: Random split data Ext : None | None | Risk Score (TRSS) |
| 69 | Gita Shafiee,2021 | Logistic Regression(Male) | AUC:0.82 (0.79–0.86) | No information | No information | Int: Random split data Ext : None | None | Formula |
| 70 | Gita Shafiee,2021 | Logistic Regression(Female) | AUC:0.87 (0.84–0.90) | No information | No information | Int: Random split data Ext : None | None | Formula |
