# Supplementary material for: Risk prediction models for sarcopenia in elderly people: a systematic review and meta-analysis
Source: Front Med (Lausanne). 2025 Jun 2;12:1589583. doi: 10.3389/fmed.2025.1589583 (PMC12171125; doi:10.3389/fmed.2025.1589583)
Supplement: Supplementary file 1 [file Data_Sheet_1.zip › Supplementary Material/Supplementary Table 2.docx]

**Supplementary Table 2**

**Bias risk and applicability assessment**

| Author,Year | Risk of Bias | | | | Applicability | | | Overall | |
| --- | --- | --- | --- | --- | --- | --- | --- | --- | --- |
|  | 1.Participants | 2.Predictors | 3.Ourcome | 4.Analysis | 1.Participants | 2.Predictors | 3.Ourcome | Risk of Bias | Applicability |
| Jiawei Chen ,2023 | - | ＋ | ＋ | - | ＋ | ＋ | ＋ | - | ＋ |
| Linlin Chen,2023 | ？ | ＋ | ＋ | - | ＋ | ＋ | ＋ | - | ＋ |
| Xi Chen,2023 | ＋ | ＋ | ＋ | - | ＋ | ＋ | ＋ | - | ＋ |
| Ting Han,2022 | - | ＋ | ＋ | - | ＋ | ＋ | ＋ | - | ＋ |
| Linghui Kong,2024 | - | ？ | - | - | ＋ | ＋ | ＋ | - | ＋ |
| Huijing Li,2023 | - | ？ | - | - | ＋ | ＋ | ＋ | - | ＋ |
| Yanping Liu,2022 | - | ？ | - | - | ＋ | ＋ | ＋ | - | ＋ |
| Yuan Zhang,2023 | - | ？ | - | - | ＋ | ＋ | ＋ | - | ＋ |
| Yibing Yue,2023 | - | ＋ | ＋ | - | ＋ | ＋ | ？ | - | ？ |
| Ying Zhang,2020 | - | ＋ | ＋ | - | ＋ | ＋ | ＋ | - | ＋ |
| Mengjuan Zhou,2023 | - | ＋ | ＋ | - | ＋ | ＋ | ＋ | - | ＋ |
| Jun-Hyun Bae,2023 | ＋ | ＋ | ＋ | - | ？ | ＋ | ＋ | - | ？ |
| Mengzhao Cui,2020 | - | ？ | - | - | ＋ | ＋ | ＋ | - | ＋ |
| Yiwen Jiang,2023 | - | ＋ | ＋ | - | ＋ | ＋ | ＋ | - | ＋ |
| Qiugui Li,2024 | - | ＋ | ＋ | - | ＋ | ＋ | ＋ | - | ＋ |
| Su Ozgur,2023 | - | ？ | - | - | ＋ | ＋ | ＋ | - | ＋ |
| Jin Ryu,2023 | ＋ | ？ | - | - | ＋ | ＋ | ？ | - | ？ |
| Yichen Yang,2023 | - | ＋ | ＋ | - | ＋ | ＋ | ＋ | - | ＋ |
| Guangjiao Yin,2023 | - | ？ | - | - | ＋ | ＋ | ＋ | - | ＋ |
| Jun-hee Kim,2024 | - | ＋ | ＋ | - | ＋ | ＋ | ＋ | - | ？ |
| Minje Seok,2023 | - | ＋ | ＋ | - | ＋ | ？ | ＋ | - | ？ |
| Minje Seok,2023 | - | ＋ | ＋ | - | ＋ | ？ | ＋ | - | ＋ |
| Keith Borges,2022 | - | ＋ | ＋ | - | ＋ | ＋ | ＋ | - | ＋ |
| Doohyun Hwang,2022 | ＋ | ＋ | ＋ | - | ＋ | ？ | ＋ | - | ？ |
| Yi-Han Mo,2022 | - | ＋ | ＋ | ？ | ＋ | ＋ | ＋ | - | ＋ |
| Shuai-Wen Huang,2023 | - | ＋ | ＋ | - | ＋ | ＋ | ＋ | - | ＋ |
| Rachel R. Deer,2020 | - | ？ | - | - | ＋ | ＋ | ＋ | - | ＋ |
| Tzyy-Guey Tseng,2020 | - | ＋ | ＋ | - | ＋ | ＋ | ＋ | - | ＋ |
| Gita Shafiee,2021 | ？ | ＋ | - | - | ＋ | ＋ | ＋ | - | ＋ |
